# Peony Seed Oil Inhibited Neuroinflammation by PPAR/RXR Signaling Pathway in D‐Gal Induced Mice

**DOI:** 10.1002/fsn3.70000

**Published:** 2025-02-27

**Authors:** Tianyu Zhang, Ying Zhang, Andong Ji, Runjia Shi, Huiying Li, Qiangcheng Zeng

**Affiliations:** ^1^ College of Life Sciences Dezhou University Dezhou Shandong China; ^2^ Institute of Nutrition and Health Qingdao University Qingdao China; ^3^ Department of Public Health The Third People's Hospital Jinan China

**Keywords:** microtubule‐associated protein tau, n‐3/n‐6 PUFAs biosynthesis, Peony seed oil, PPAR/RXR signaling pathway

## Abstract

Essential fatty acids could regulate inflammation, especially n‐3 PUFA (n‐3 polyunsaturated fatty acids), which are considered to have a protective effect to inhibit neuroinflammation. Peony seed oil is one of the most abundant n‐3 PUFAs in oils. but the mechanism of peony seed oil affecting inflammation in mice brains is still lacking convincing evidence. Sixty male C57BL/6J mice were randomly allocated into four groups: D‐gal (D‐galactose) induced model group, FO (D‐gal + fish oil), PSO (D‐gal + peony seed oil). After 10 weeks, the fatty acid composition in liver and brain tissues and potentially related genes were examined. Docosahexaenoic acid (DHA) was significantly higher, while arachidonic acid (AA) was significantly lower in both in the PSO and FO groups than that in the model group in the brain and liver. In the PSO and FO groups, the relative mRNA levels of *Fads1/2*, *Elovl2*, and *Acaa1a* were significantly up‐regulated, but *Acox1* and *Acox3* were significantly down‐regulated compared to the model group. In the PSO and FO groups, the relative protein levels of PPARG, RXRA, and IL‐10 were significantly up‐regulated, and the expressions of AGERs, TNF‐α, PLA2, and PGF2α were significantly down‐regulated compared to the model group. The phosphorylation‐tau of total tau protein ratio was significantly lower in the PSO and FO groups than in the model group. Peony seed oil, rich in n‐3 PUFA, inhibited neuroinflammation and rescued the disruption of alternative splicing of the *Mapt* gene by activating the PPAR/RXR signaling pathway and promoting n‐3/n‐6 biosynthesis.

## Graphical abstract

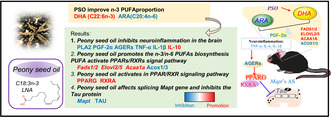

**Abbreviations**
AA: Arachidonic acid
ACAA1A: A acyltransferase 1A
ACOX1/3: Acyl‐Coenzyme A oxidase
AGERs: Advanced Glycosylation End‐Product Specific Receptor
ALA: α‐linolenic acid
APP: Amyloid Precursor Protein
AS: alternative splicing
BBB: blood–brain barrier
D‐gal: D‐galactose
DHA: Docosahexaenoic acid
ELOVL2: very long fatty acid protein 2
FADS1/2: fatty acid desaturase 1/2
GO: gene ontology
IL‐10: Interleukin 10
IL‐1β: Interleukin 1beta
KEGG: Kyoto Encyclopedia of Genes and Genomes
MAPT: Microtubule Associated Protein Tau
MFSD2A: Major Facilitator Superfamily Domain Containing 2A
MXE: mutually exclusive exons
PGF2α: Prostaglandin F2 alpha
PLA2: Phospholipase A2
PPARs: Peroxisome proliferators‐activated receptors
PSO: Peony seed oil
PUFAs: Polyunsaturated fatty acids
RA: Retinoic acid
RBP4: Retinol‐binding protein 4
RXRs: Retinoid X receptors
TNF‐2α: tumor necrosis factor alpha

## 1 Introduction

The daily diet is inseparable from the intake of essential fatty acids. Docosahexaenoic acid (C22:6n‐3, DHA) and arachidonic acid (C20:4n‐6, AA), as the main components of polyunsaturated fatty acids (n‐3 PUFA), and n‐6 PUFAs, respectively, often need dietary intake of α‐linolenic acid (C18:3n‐3, ALA) and linolenic acid (C18:2n‐6, LA) to make up (Harayama and Shimizu 2020). “Oil peony” means that peony could be used as raw materials to prepare peony seed oil because of its nutrients (mainly rich in ALA) and has attracted much attention due to its potential applications. In our previous research, we found that peony seed oil supplementation can improve cognitive function by increasing n‐3 PUFAs in the brain, reducing D‐gal‐induced neuronal apoptosis, and improving cognitive function (Zhang et al. 2022). However, its basic research is far less than that of flaxseed oil, perilla oil, and other plant‐based n‐3 PUFAs. But the biosynthesis, elongation, and beta‐oxidation of ALA and LA are the same, with DHA‐mediated inhibition of LA metabolism through ∆‐6 and ∆‐5 desaturases or inhibition of AA elongation (Jalil et al. 2019; Hishikawa et al. 2020). Generally, the content of DHA and ALA is relatively stable in tissues, such as the liver, brain, and heart; the proportion of DHA and AA in components of membrane phospholipids plays a beneficial role in the biosynthesis and metabolism of fatty acids. The proportion and balance of n‐3 PUFA and n‐6 PUFAs are related to the maintenance of body function and the occurrence of diseases.

D‐gal (D‐galactose) administered has been found to induce aging and cognitive function decline in a variety of rodent models, the most significant being neurodegeneration and neuroinflammation (Xiao et al. 2011; Abd El‐Fatah et al. 2021). Cognitive function decline is widely believed to be related to the aggregation of β‐amyloid peptide 1–42 (Aβ42) and hyperphosphorylated tau to form senile plaques and neurofibrillary tangles. Although DHA in the brain is critical for learning and memory and positively affects cognitive function, DHA's effect on tau protein was not clear (Belviranlı and Okudan 2019; Zhu et al. 2020). However, the ability to transport DHA in aging or Alzheimer's disease (AD) animal models that have been impaired may be related to n‐6 PUFA intake, excessive induced desaturases, and lipid peroxidation. Some researchers believe that dietary fish oil rich in DHA improves AD pathology and reduces AD risk (Román et al. 2019). After the biosynthesis of DHA in the liver, free DHA is transported through the blood–brain barrier (BBB) via passive diffusion. Supplementation of oil rich in DHA or ALA will restore the ratio of DHA, which has a positive effect on cognitive function (Ouellet et al. 2009).

DHA could also act as a molecule of retinoid X receptors (RXR), which form heterodimers with peroxisome proliferator‐activated receptors (PPARs) and retinoic acid receptors (RARs), which are known for their functions in lipid metabolism and inflammation. PPAR has three isoforms in the brain and other organs: PPARα (PPARA), PPARβ (PPARD), and PPARγ (PPARG). According to the activation of ligands, each isoform has its tissue expression and different physiological functions. PPAR signaling pathway plays a significant role in inflammation, fatty acid metabolism, insulin resistance (IR), and cognitive function (van Neerven, Kampmann, and Mey 2008). In addition, PPARα regulates the expression of genes encoding enzymes involved in the metabolism of amyloid precursor proteins (APPs) and down‐regulates β‐secretase (BACE‐1). PPAR agonist activates all PPAR receptors and significantly decreases Aβ plaques of Aβ level and microglia activation in AD model mice (Kummer et al. 2015; Chang et al. 2019). Nowadays PPARs seem to be a promising target in AD prevention. However, there is still insufficient evidence to show the molecular mechanism of n‐3 PUFA affecting Tau protein.

In the current study, both fish oil and peony seed oil are rich in n‐3 PUFA, but how to regulate DHA and AA's metabolism and biosynthesis‐related enzymes is unknown. Deeply RNA sequencing is an important method applied in gene regulation in neural neurophysiology, brain science, and other research areas (Wang et al. 2008; Wang, Gerstein, and Snyder 2009). Alternative splicing (AS) produces the complexity of the transcriptome and proteome in higher organisms. AS' change is the basis of gene regulation in biological and disease processes (Kalsotra and Cooper 2011; Stilling et al. 2014). The current research aims to find convincing evidence of a relationship between n‐3 PUFA supplements and neuroinflammation, even the mechanism of interaction with Tau protein.

## 2 Materials and Methods

### 2.1 Animals and Experiment Design

Sixty male C57BL/6J mice were obtained from SPF Biotechnology Co. Ltd. (Beijing, China). (licensed ID: SCXK 2019‐0010). Then animals were divided into four mice per cage and housed for 12 h:12 h light–dark cycles with a normal chow diet. C57BL/6J mice were randomly allocated into six groups: FO (D‐gal + fish oil), PSO (D‐gal + peony seed oil), CO (D‐gal + corn oil), OO (D‐gal + olive oil), LO (D‐gal + lard oil), and the model group, respectively. Then killed and collected tissue after 10 weeks of feeding. All experimental procedures were approved by the Institutional Animal Care at Qingdao University (QDU‐AEC‐2023432).

### 2.2 Fatty Acid and Fat‐Soluble Vitamins Detection Analysis

The detailed method of the fatty acid composition refers to the previous method by gas chromatography (GC) (Agilent 7890A, USA). Fatty Acid Methyl Ester standards mix (FAME) (CRM47885, Supelco, USA) as a reference (Cai et al. 2020). Fat‐soluble vitamin standards were mixed and determined by high‐performance liquid chromatography (HPLC) (Agilent 1200, USA).

### 2.3 RT‐qPCR Analysis

Total RNA of brains and livers was extracted by TRIzol and reversed transcribed into a cDNA with a cDNA synthesis kit. Quantitative PCR was performed using SYBR green with the QuantStudio 1 PCR System (A40425, ThermoFIsher, USA) following the previous method (Zhang et al. 2019). Then the relative expression was calculated as $2^{\Delta \Delta C_{t}}$ = $2^{\Delta \left(\Delta C_{t}\;\text{Target gene}-\Delta C_{t}\;\text{Actb}\right)}$, The *Actb* gene was used as a reference. The target gene's primer information was shown in Table S1.

### 2.4 Western Blot

Brain tissue was rinsed in pre‐cooling PBS, and about 50 mg was added to RIPA lysis buffer to extract total protein brain samples, and western blot (WB) analysis was performed. Firstly, the homogenate was cracked and centrifuged. Combined with PVDF membrane by SDS‐PAGE electrophoresis After which the membrane was incubated with the primary antibody PPARG (A11183 Abclonal, WuHan, China) and RXRA (A15242) in a 4°C refrigerator overnight. On the second day, TBST was washed and then incubated with the second antibody at room temperature for 2 h. ActB (AC038) was used as the control reference protein and normalized. According to the previous analysis methods, ImageJ software was used to analyze the relative expression levels of the detected proteins (Zhang et al. 2022).

### 2.5 RNA‐Seq and Differential Gene Expression Analysis

Brain's RNA samples were conducted on the Illumina HiSeq 2000 platform, and then quantity control of the sequence was performed using FastQC v0.10 (de Sena Brandine and Smith 2019). Clean reads were sequenced to mouse reference and obtained the location information by STAR 2.5.3a software (Dobin et al. 2013). RNAseq data were uploaded to the National Genomics Data Center (NGDC) (https://ngdc.cncb.ac.cn/) (CNCB‐NGDC Members and Partners 2021) and the BioProject number is CRA004459. Differential gene expression was determined using DESeq2. The *p*‐value was corrected by Benjamin & Hochberg (BH) multiple tests to get the *Q*‐value (adjusted *p*‐value) < 0.05, and |FoldChange|> 1.5 (Love, Huber, and Anders 2014).

The R Bioconductor/cluster_profile and Metascape (http://metascape.org) were applied for Gene Ontology (GO), and the Kyoto Encyclopedia of Genes and Genomes (KEGG) has been previously described (Wang, Wang, and Li 2012; Wang et al. 2020). The protein–protein interaction (PPI) network was visualized with Cytoscape (v3.1.2).

### 2.6 Transcription Factor Prediction and Multivariate Analysis of Transcript Splicing

We used RNA‐seq to screen differential transcription factors (TFs), combined with UCSC Genome Browser (http://genome.ucsc.edu/) and JASPAR 2020 (http://jaspar.genereg.net/) to analyze the confidence and correlation (Fornes et al. 2020). Then, alignment files (Bam) and reference annotations (GTF) were passed to rMATS software, which is a computational tool to detect differential AS events from RNA‐Seq data. Differential expression of alternative splice was determined using Junction Counts, significant DAGs|IncLevelDifference|> 0.01 and FDR < 0.05 (Shen et al. 2014).

### 2.7 Enzyme‐Linked Immunosorbent Assay

Brain was homogenized in 50 mg tissue/mL cold PBS and was centrifuged at 12,000 *g* for 15 min, and protein was detected using the BCA method. According to the ELISA kit manufacturer's protocol to measure the level of TNF‐α, AGERs, BDNF, IL‐1β, IL‐10, and PGF2α (Mlbio, Shanghai, China) in serum and brain.

### 2.8 Statistical Analysis

All experiments were repeated at least three times, and data were performed as means with standard deviation (mean ± SD). All correlations were calculated using Pearson's correlation coefficient, respectively. One‐way analysis of variance (ANOVA) followed by *t*‐test using Graph‐Pad Prim software is considered significantly different if *p*‐value < 0.05 (*) or *p*‐value < 0.01 (**).

## 3 Results

### 3.1 Peony Seed Oil Inhibits Neuroinflammation in the Brain

**FIGURE 1:**
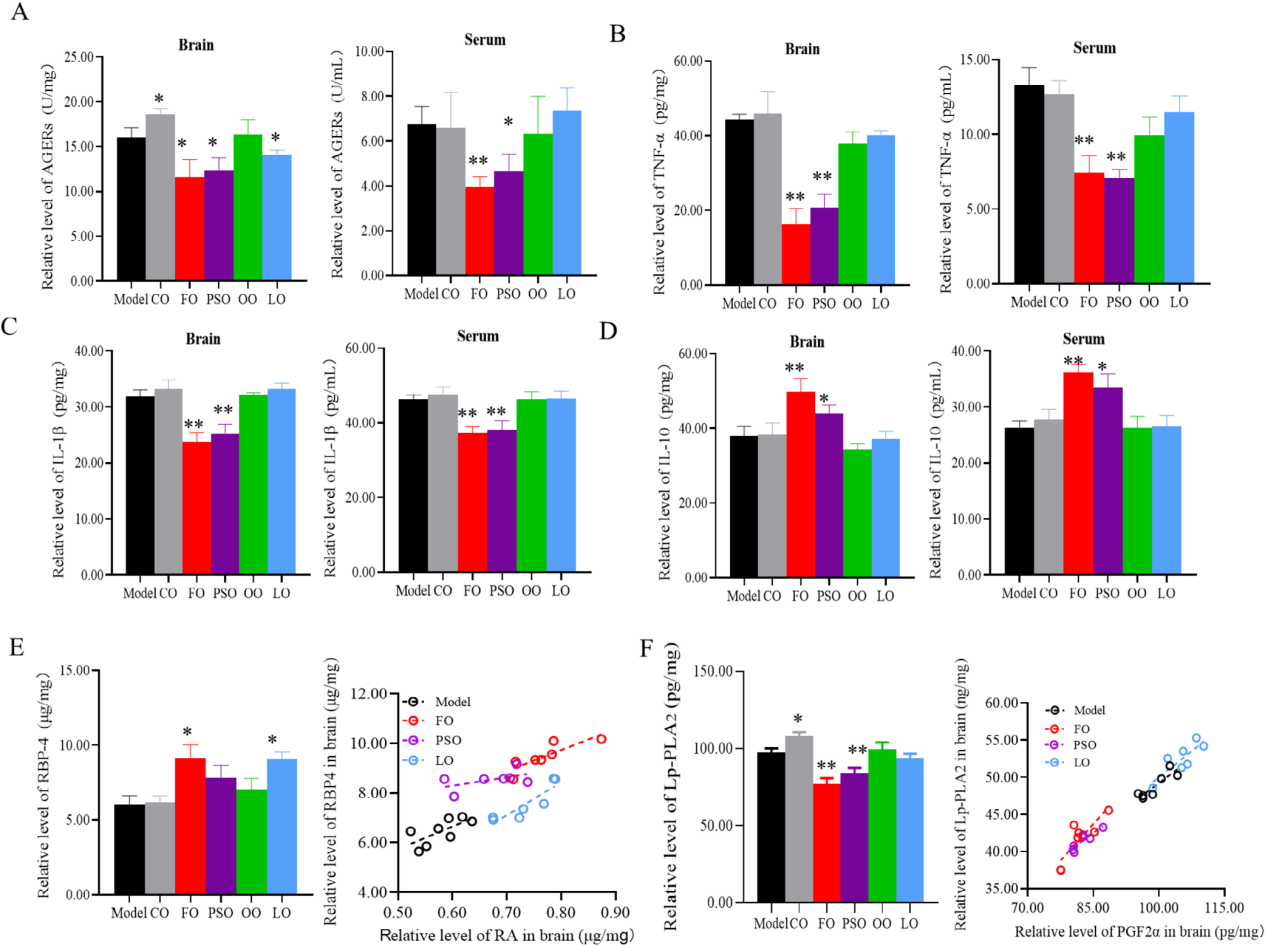
Peony seed oil inhibits neuroinflammation. (A) Determination of serum and brain homogenate concentrations of AGERs assessed by ELISA. (B–D) Determination of concentrations of pro‐inflammatory cytokines (IL‐1β, TNF‐α) and anti‐inflammatory cytokines (IL‐10) in serum and brain homogenate assessed by ELISA. (E) Relative of RBP4's content with corresponding RA's content. (F) The relative level of Lp‐PLA2 with corresponding PGF2α's content. Data are presented as mean ± SD; *p < 0.05. **p < 0.01, a significant difference compared to the model group determined by the *t*‐test. Pearson's correlation is a measure of a linear correlation in the data. Three biologically independent samples per group.

We first recorded that there were no significant differences in feed intake and body weight among the groups (Figure S1A). Then we found that PSO, FO, and LO inhibited the values of AGERs, which is harmful to inflammation induced by D‐gal (Figure 1A; *p* < 0.05). To investigate the effect of peony seed oil and fish oil on neuroinflammation, we detected the pro‐inflammatory and anti‐inflammatory cytokines in brain homogenate and serum; the concentration of TNF‐α and IL‐1β was significantly decreased in the PSO and FO groups compared to the model group, but the concentration of IL‐10 was significantly increased in the PSO and FO groups compared to the model group (Figure 1B–D; *p* < 0.05).

We found that the concentration of retinol‐binding protein 4 (RBP4), which is used to bind blood‐derived retinyl esters and retinol (Klöting et al. 2007). Retinol acid (RA)’ increases were significant differences and correlations with RBP4's concentration in the FO group (*R* ^2^ = 0.711; *p* = 0.017) and LO group (*R* ^2^ = 0.787; *p* < 0.008) (Figure 1E; *p* < 0.05). Meanwhile, PGF‐2α, as an important metabolite of AA, its increase was a significant difference and correlation with Lp‐PLA2's concentration in the FO group (*R* ^2^ = 0.743; *p* = 0.013) and PSO groups (*R* ^2^ = 0.805; *p* = 0.006) compared to the model group (*R* ^2^ = 0.783; *p* = 0.008) (Figure 1F; *p* < 0.05). In this part, we found that n‐3 PUFA could inhibit neuroinflammation by inhibiting AA metabolism and promoting RA metabolism.

### 3.2 Peony Seed Oil Promotes the n‐3/n‐6 PUFAs Biosynthesis

First of all, no changes in liver coefficient among the groups. Then the liver's fatty acid composition was detected because both of them are the most important organs in the process of fatty acid synthesis, metabolism, and transportation (Pawlosky, Barnes, and Salem 1994). Compared with the model group, the proportion of DHA was significantly higher in the PSO and FO groups, and the proportion of AA was significantly lower in the PSO and FO groups. However, there was no significant change in the proportion of palmitic acid (PA, C16:0), palmitoleic acid (POA, C16:1n‐7), stearic acid (SA, C18:0), and oleic acid (OA, C18:1n‐9) in the liver (Table 1; *p* < 0.05).

**TABLE 1:** Fatty acid composition in liver.

% Fatty acid	Model (*N* = 6)	CO (*N* = 5)	FO (*N* = 6)	PSO (*N* = 5)	OO (*N* = 6)	LO (*N* = 5)
Liver						
C16:0	23.12 ± 1.52	22.25 ± 0.86	20.85 ± 1.47	20.76 ± 1.28	21.22 ± 1.72	22.15 ± 1.52
C18:0	22.26 ± 1.73	20.85 ± 1.75	20.53 ± 1.32	20.03 ± 1.47	20.02 ± 1.72	20.12 ± 1.55
Total SFA	46.21 ± 1.56	45.56 ± 1.66	43.56 ± 1.39	43.52 ± 1.25	44.56 ± 1.69	44.63 ± 1.43
C16:1n‐7	2.76 ± 1.31	2.56 ± 1.35	2.23 ± 1.52	3.47 ± 1.41	4.02 ± 1.45	4.11 ± 1.37
C18:1n‐9	14.42 ± 1.43	14.13 ± 1.05	15.52 ± 1.29	16.18 ± 1.74	16.44 ± 1.35	16.82 ± 1.41
Total MUFA	16.85 ± 1.21	17.56 ± 1.11	17.45 ± 1.23	19.33 ± 1.65	19.85 ± 1.31	19.82 ± 1.36
C18:2n‐6	2.25 ± 0.27	2.56 ± 0.22	3.13 ± 0.54	2.96 ± 0.34	3.56 ± 0.63	3.85 ± 0.48
C18:3n‐3	0.25 ± 0.08	0.22 ± 0.09	0.34 ± 0.12	0.32 ± 0.08	0.27 ± 0.10	0.24 ± 0.08
C20:4n‐6	18.60 ± 0.97	18.46 ± 1.38	14.54 ± 1.39*	14.21 ± 1.27**	17.73 ± 1.58	18.08 ± 1.55
C20:5n‐3	1.56 ± 0.47	1.17 ± 0.27	1.89 ± 0.27	1.88 ± 0.27	1.56 ± 0.27	1.50 ± 0.27
C22:4n‐6	3.01 ± 0.96	3.22 ± 0.86	2.86 ± 0.74	2.88 ± 0.56	3.11 ± 0.41	3.18 ± 0.86
C22:6n‐3	9.04 ± 1.03	9.30 ± 0.98	14.11 ± 0.69**	13.35 ± 1.01**	10.29 ± 1.25	10.42 ± 0.88
Total PUFA	34.52 ± 1.49	33.43 ± 1.19	34.76 ± 1.52	34.82 ± 1.23	34.04 ± 1.45	34.85 ± 1.51
Total n‐3 PUFA	10.52 ± 1.03	10.37 ± 1.09	15.86 ± 1.56**	15.56 ± 1.43**	12.56 ± 1.31	12.50 ± 1.16
Total n‐6 PUFA	24.52 ± 1.17	24.96 ± 1.44	19.66 ± 1.33**	19.98 ± 1.36**	24.86 ± 1.16	24.31 ± 1.80
C16:1n‐7/C16:0	0.18 ± 0.03	0.16 ± 0.03	0.18 ± 0.03	0.18 ± 0.05	0.22 ± 0.04	0.21 ± 0.04
C18:1n‐9/C18:0	0.65 ± 0.08	0.68 ± 0.07	0.74 ± 0.06	0.75 ± 0.05	0.77 ± 0.04	0.78 ± 0.06
C22:6n‐3/C20:4n‐6	0.47 ± 0.05	0.51 ± 0.04	0.65 ± 0.05**	0.63 ± 0.04**	0.41 ± 0.04	0.38 ± 0.05
n‐3/n‐6 PUFA	0.52 ± 0.08	0.51 ± 0.06	0.88 ± 0.05**	0.85 ± 0.06**	0.56 ± 0.07	0.59 ± 0.04

*Note:* Result's are expressed as (mean± SD) for per group (*N* =5 or 6 per group). Significant difference from other groups compared to the Model group (* *p* < 0.05 or ** *p* < 0.001) determined by Ony‐way ANOVA followed T‐test.

**FIGURE 2:**
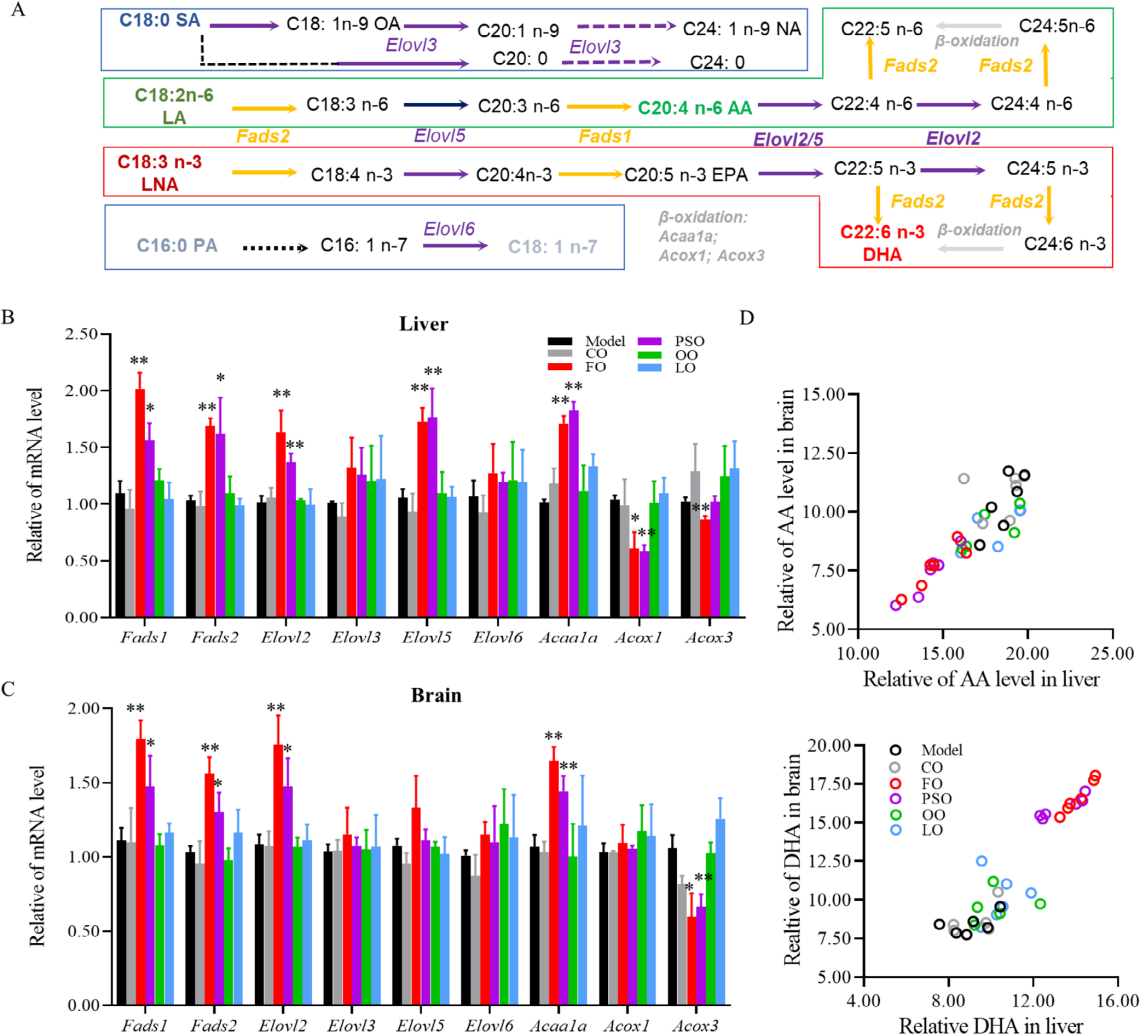
Peony seed oil promotes the n‐3/n‐6 PUFA biosynthesis. PUFAs/MUFAs biosynthesis pathway in liver and brain. Fatty acid desaturation enzymes are shown in yellow. Fatty acid elongation and enzymes are shown in purple. β‐oxidation and enzymes are shown in gray. (B, C) qRT‐PCR analysis of the PUFAs/MUFAs biosynthesis‐related genes in liver and brain. (D) Correlation of DHA, AA proportion in the liver with the brain. Data are presented as mean ± SD; *p < 0.05. **p < 0.01, a significant difference compared to the model group determined by the *t*‐test. Pearson's correlation is a measure of a linear correlation in the data. Three biologically independent samples per group.

So we investigated the critical enzymes in the process of long‐chain fatty acid (LCFA) biosynthesis, elongation, and β‐oxidation in the peroxisome, such as fatty acid desaturase (FADS1/2) and elongation of very long fatty acid protein (ELOVL2/3/5/6), and β‐oxidation in peroxisome requires acetyl‐Coenzyme A acyltransferase 1A (ACAA1A) and acyl‐Coenzyme A oxidase (ACOX1/3) (Figure 2A). We found that the expression of *Fads1*, *Fads2*, *Elovl2*, *Elovl5*, and *Acaa1a* was significantly higher, and the expression of *Acox1* and *Acox3* was significantly lower in both the PSO and FO groups compared to the model group (Figure 2B; *p* < 0.05). While in the brain, the expression of *Fads1*, *Fads2*, *Elovl2*, *and Acaa1a* was significantly higher but the expression of *Acox3* was significantly lower in both PSO and FO groups compared to the Model group (Figure 2C; *p* < 0.05). In addition, there was a significant correlation between the liver and brain increase of DHA in PSO group (*R* ^2^ = 0.897; *p* < 0.01) and FO group (*R* ^2^ = 0.972; *p* < 0.01). Meanwhile, AA's decrease between the liver and brain has a significant correlation in Model group (*R* ^2^ = 0.870; *p* < 0.01), CO group (*R* ^2^ = 0.801; *p* < 0.05), FO group (*R* ^2^ = 0.848; *p* < 0.01) and PSO group (*R* ^2^ = 0.926; *p* < 0.01) (Figure 2D; *p* < 0.05). In the section, peony seed oil and fish oil supplements improved the n‐3/n‐6 PUFAs biosynthesis and elongation in the liver and brain.

### 3.3 Peony Seed Oil and Fish Oil Affect Fatty Acid Metabolic/Transport‐Related Gene Function

**FIGURE 3:**
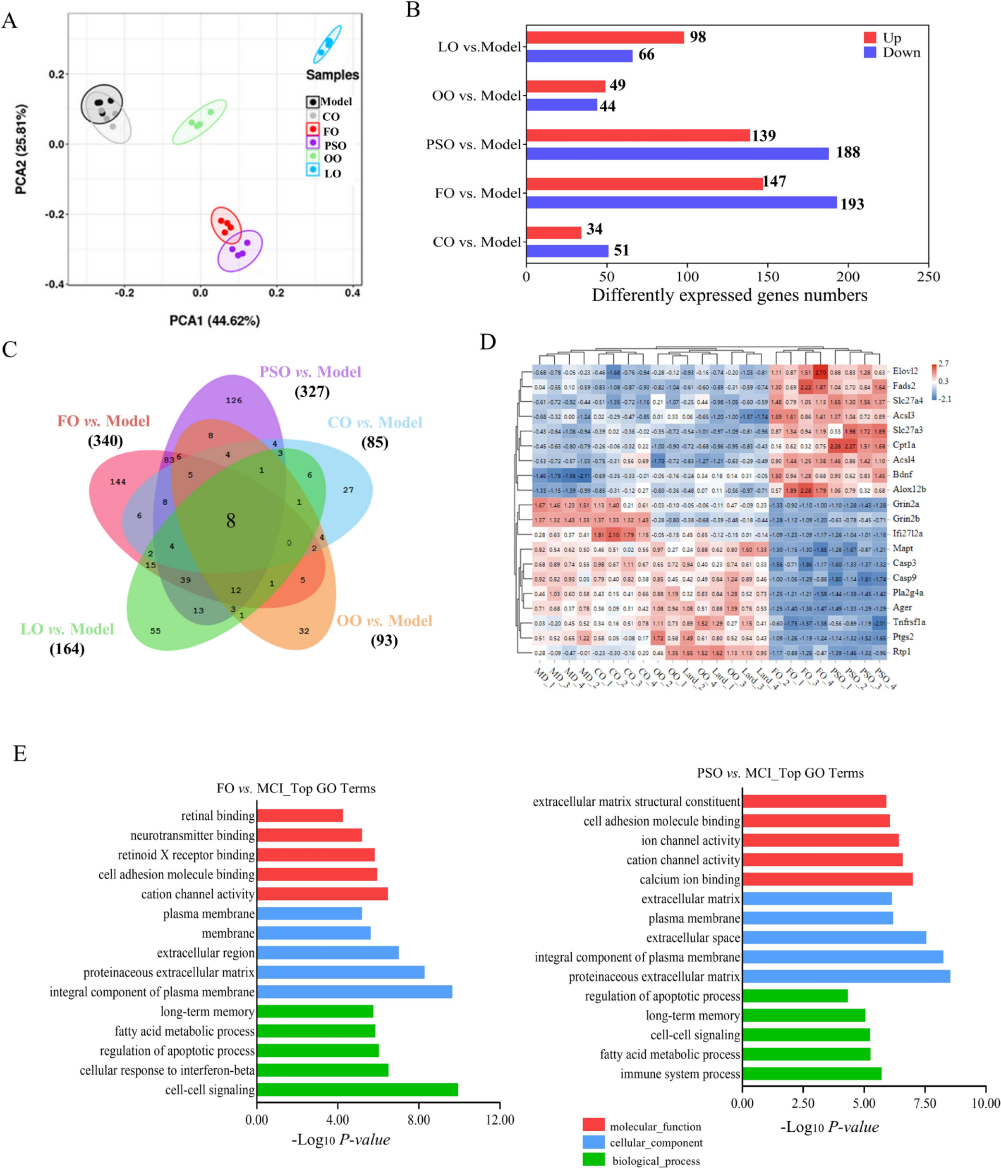
RNA‐seq analysis showed Peony seed oil affects fatty acid metabolic/transport‐related gene function. (A) Principal component analysis (PCA) showed the PSO group (purple) and FO group (red)'s samples were completely segregated from the other groups. (B) Up‐regulated DEGs (red) and down‐regulated DEGs (blue) in CO, OO, FO, PSO, and LO groups compared to the model groups. (C) The number of DEGs in the different groups are presented as Venn diagrams. (D) Genes related to the fatty acid metabolic/transport process were represented by a heatmap. (E) Top GO terms of biological process (BP), molecular function (MF), and cellular component (CC) in FO and PSO groups versus the model group. bars represent the −log_10_ (*p*‐values). DEGs were identified by *p*‐value < 0.05, |Log_2_Foldchange| > 1. Data are representative of four replicates that were sequenced, respectively.

To find out the relationship of n‐3 PUFA biosynthesis, metabolism, and detailed mechanism, RNA‐seq analysis of the brain helped us find that PSO, and FO groups were well differentiated from the model group (Figures 3A and S2A). Compared with the model group, a total of 34, 49, 98, 188, and 193 DEGs were up‐regulated, and a total of 51,44, 66, 139, and 147 DEGs were down‐regulated in CO, OO, LO, PSO, and FO groups, respectively (Figures 3B and S2B–F; *p* < 0.05), A total eight common DEGs in the CO, OO, LO, PSO, and FO groups were compared to the model group (Figure 3C,D).

GO functional analysis in intervention groups compared to the model group could be divided into two levels; we found that in PSO and FO‐vs‐model, it mainly involves cell–cell signaling, cell adhesion, fatty acid metabolism process, regulation of apoptotic process, and long‐term memory of the biological process. In cellular components, it mainly affects the membrane, an integral component of the plasma membrane. In molecular function, cellular response to retinoic acid and retinoid X receptor (RXR) binding. (Figures 3E and S3A,B; *p* < 0.05).

### 3.4 Peony Seed Oil Activates in PPAR/RXR Signaling Pathway

**FIGURE 4:**
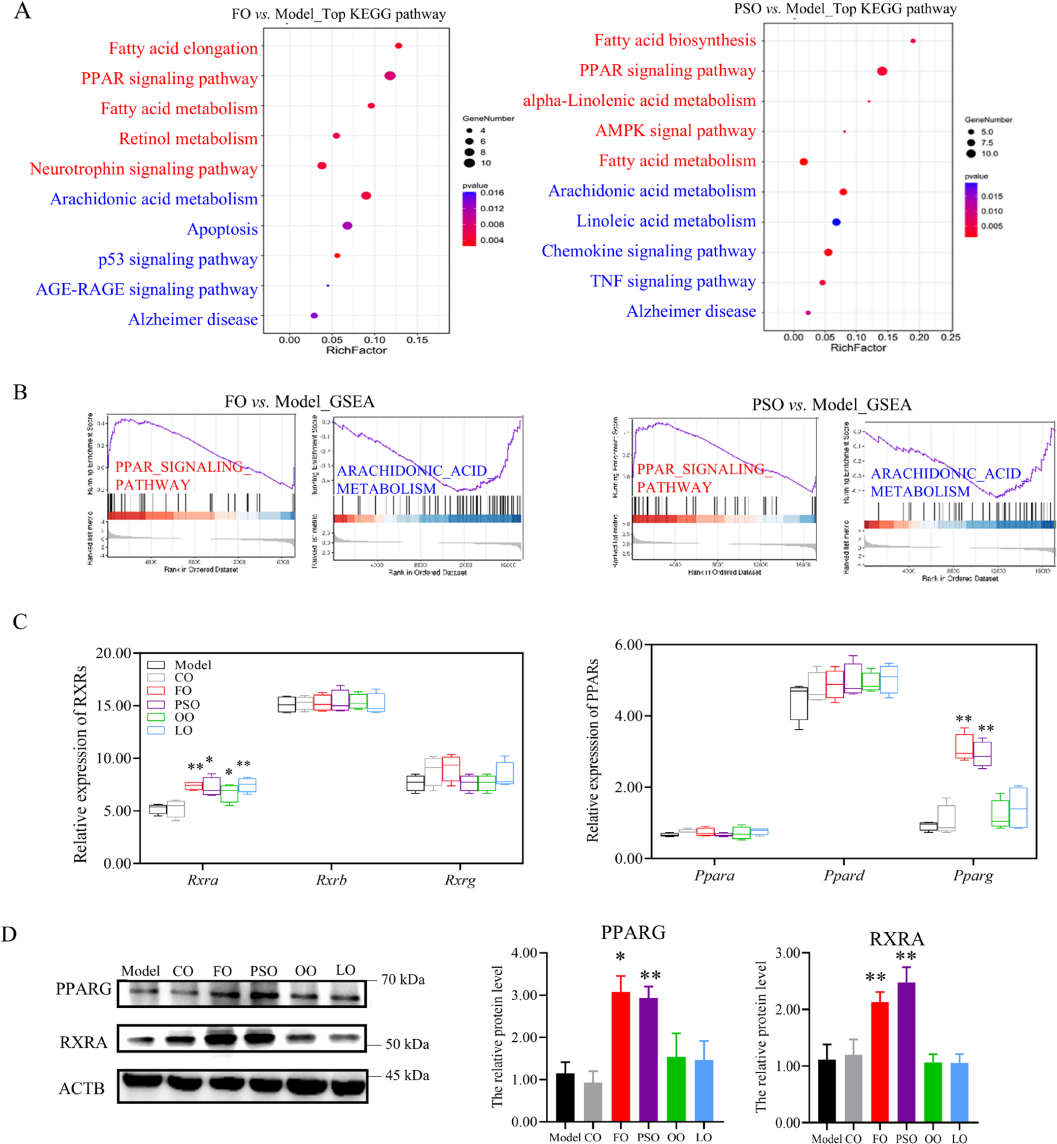
Peony seed oil activates the PPAR/RXR signaling pathway. (A) Up‐regulated (red) and down‐regulated (blue) top pathways in FO and PSO groups versus the model group by KEGG analysis. The size of the plots indicates the DEG numbers, and color indicates the *p*‐values enriched in the pathways. (B) Target genes enriched in up‐regulated (red) or down‐regulated (blue) pathways by GSEA. (C) The expression of the PPARs/RXRs family. bars represent the log_10_ (FPKM). (D) WB detection of relative PPARG and RXRA protein levels. ActB was used as a loading control for reference. Data are presented as mean ± SD; *p < 0.05. **p < 0.01, a significant difference compared to the model group determined by the *t*‐test. Three biologically independent samples per group.

Then, in the DEGs enrichment analysis by KEGG and GSEA, we found that in PSO, FO, and up‐regulated PPAR signaling pathways and down‐regulated arachidonic acid metabolism. In the PSO‐vs‐model group, up‐regulated in alpha‐linolenic acid metabolism and Fatty acid biosynthesis, down‐regulated Linoleic acid and TNF signal pathway. While in the FO‐vs‐model group, up‐regulated retinol metabolism and fatty acid elongation, down‐regulated AGE‐RAGE signaling pathway, and apoptosis. (Figures 4A,B and S4A and S3B; *p* < 0.05).

Then we investigated the main TFs of PPARs and RXRs; both of them have three isoforms in the brain, respectively. We find that *Pparg* ‘s gene expression was significantly higher in the PSO and FO groups compared to the model group. The *Rxra*'s gene expression was higher in the PSO, FO, OO, and LO groups compared to the model group (Figure 4B; *p* < 0.05). Then, the relative protein level of PPARG was also significantly higher in the PSO and FO groups than in the model group. the relative protein level of RXRA was significantly higher in the PSO, FO, OO, and LO groups compared to the model group (Figure 4C,D; *p* < 0.05). So we found that the n‐3 PUFA supplement activates the PPAR/RXRs signaling pathway.

### 3.5 Peony Seed Oil Affects Splicing of the *Mapt* Gene and Inhibits the Tau Protein

**FIGURE 5:**
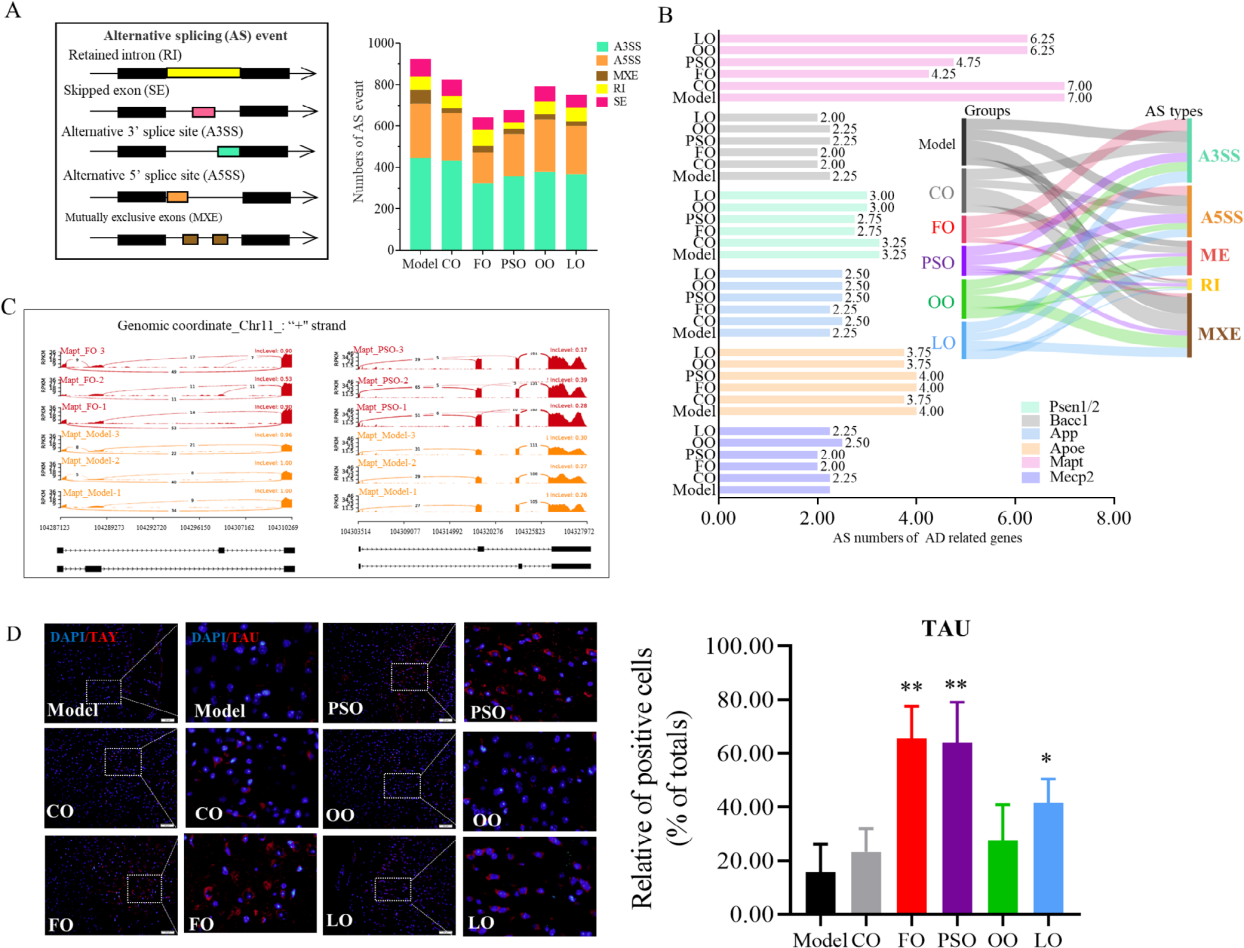
Peony seed oil affects splicing *Mapt* and inhibits the Tau expression. (A) Column charts highlight the number and frequency of distinct alternative splicing events detected using rMATS. (B) The AS numbers of AD risk factors' genes were screened. (C) Per‐base expression is plotted on the y‐axis of the Sashimi plot, genomic coordinates on the x‐axis, and mRNA isoforms quantified are shown on the bottom (exons in black, introns as lines with arrowheads). FO and PSO groups colored red, model group colored yellow. (D) Representative immunofluorescent images of TAU (red) positive cells in the cortex. DAPI (blue) was used for nuclei staining. Scale bar, 50 & 100 μm. Analysis of the positive cells (% total cells) in the cortex. Ten or three independent samples per group, respectively. Data are presented as mean ± SD; *p < 0.05. **p < 0.01, a significant difference compared to the model group determined by the *t*‐test. Three biologically independent samples per group.

Aim to further explore the regulatory mechanism and correlation of n‐3 PUFA on key risk factors of differential AS, which is recognized as a regulator of protein structure and function. According to the types of AS, it can be divided into alternative 5′ splice site (A5SS), alternative 3′ splice site (A3SS), mutually exclusive exons (MXE), and skipped exons (SE), and intron retention (IR) by rMATS (Hsieh et al. 2019). We found that the numbers of AS events were lower in the PSO and FO groups compared to the model group (Figure 5A; *p* < 0.05). Next, aim to find whether the types and numbers of AS are related to the key risk genes in AD, including *Psen1/2*, *Bace1*, *App*, *Apoe*, *Mecp2*, and *Mapt*. Most notably, the AS numbers of the *Mapt* gene, which is located on chromosome 11 and mainly functional, were encoded by tau protein and were decreased in the PSO and FO groups, and the main AS type is MXE (Figure 5B,C). Then, we want to investigate the changes of tau protein and phosphorylation when the AS state of the exon near the *Mapt* gene promoter region changes. Then, the TAU's protein expression in the PSO, FO, and LO groups was significantly higher than in the model group (Figure 5D; *p* < 0.05). The series of evidence indicated that n‐3 PUFA disturbed splicing *Mapt* and inhibited Tau protein expression.

## 4 Discussion

In our research, a daily diet that has a certain proportion of n‐3 PUFA, such as peony seed oil rich in ALA or directly supplementing with fish oil rich in DHA, both have anti‐inflammatory and neuroprotective effects on activating the PPARs/RXRs signaling pathway. As is known to us, the PPAR family regulates several metabolic and cellular processes in organisms, including energy homeostasis related to lipid and glucose metabolism, fat production, inflammatory response, or oxidative stress (Echeverría et al. 2016). In our study, we found that adequate intake of ALA or its derived DHA + EPA can maintain PPARG activity and play an important neuroprotective role in brain tissue. In addition to ALA and LA mentioned in this article, stearidonic acid (C18:4n‐3, SDA) and γ‐linolenic acid (C18:3n‐6, GLA) are also important fatty acids in the body, especially in mammals. However, the proportion of these two fatty acids in brain tissue is almost undetectable. In addition, the imbalance of n‐6 and n‐3 PUFA levels in the organization affects the biosynthetic pathway, as LA and ALA compete for the same enzyme mechanism (Videla et al. 2022). The specific mechanism is in the fact that the n‐3 PUFA has a positive impact on the biosynthesis and metabolism of DHA. On the other hand, FADS1, FADS2, and ELOVL2 have higher affinity for n‐3 PUFA compared to n‐6 PUFA in the liver, while there is no significant difference between various enzymes in the brain (Valenzuela et al. 2014). AA's biosynthesis and metabolism processes were inhibited by increasing *Fads2*, *Elovl2*, *and Acaa1a* and decreasing *Acox1*. BBB prevents harmful molecules from entering the brain from the blood, but it also prevents about 98% of small molecule drugs, which is also an important reason for the limited treatment of neurodegenerative diseases, stroke, and other diseases (Ben‐Zvi et al. 2014). When DHA enters the BBB competitively in the form of LPC‐DHA, mainly by inhibiting the PLA2's expression. DHA could also inhibit D‐gal‐induced neuroinflammation, mainly by inhibiting AGERs, TNF‐α, and IL‐1β, promoting the anti‐inflammation factors of IL‐10's expression. If the balance between DHA and AA's metabolism and biosynthesis had been broken, enrichment of DEGs showed that peony seed oil and fish oil supplements could activate *Pparg* and *Rxra*, both of which are major TFs. The RBP4's expression and transformation were correlated with RA concentration in the brain.

**FIGURE 6:**
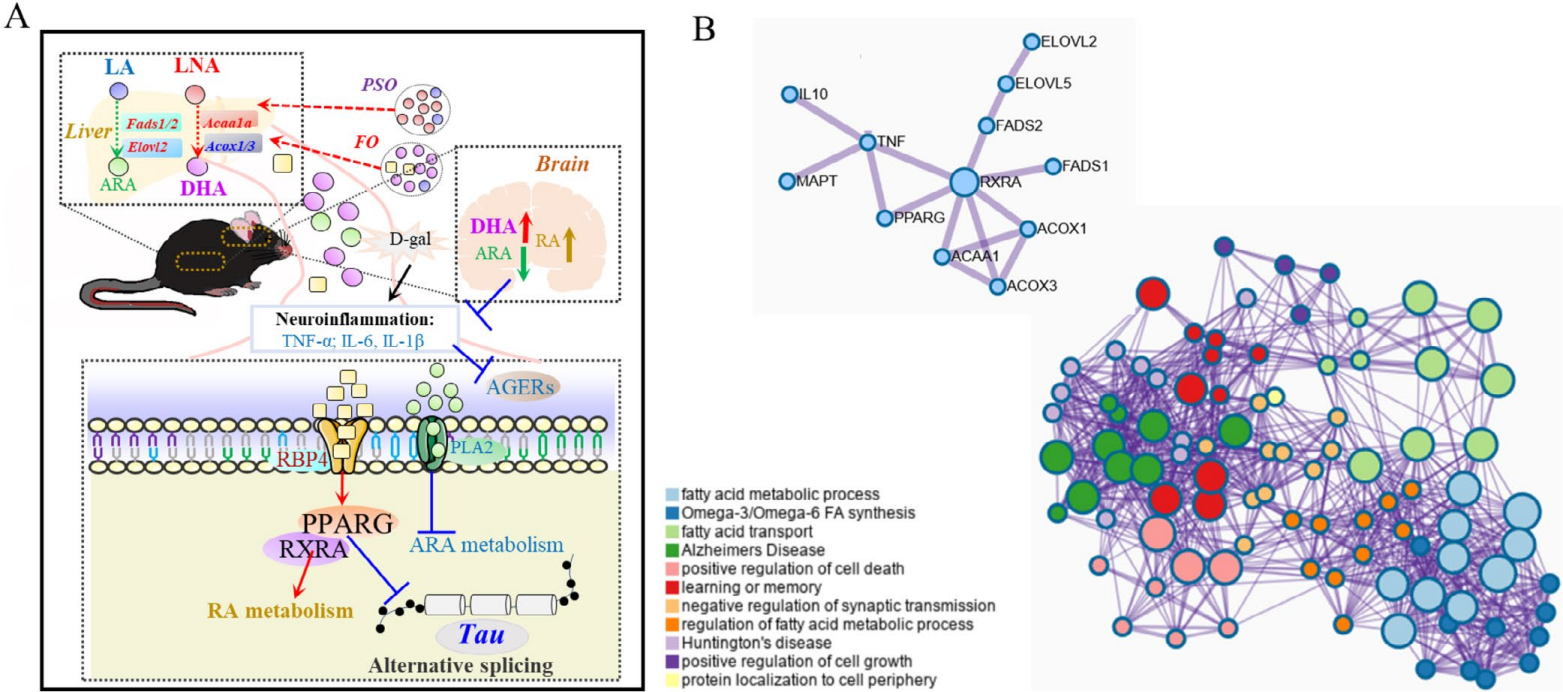
Potential mechanism of peony seed oil in liver and brain induced by D‐gal. (A) Peony seed oil and fish oil inhibited neuroinflammation and hyperphosphorylation of Tau protein by activating the PPARs/RXRs signaling pathway. (B) Enrichment analysis of DEGs was represented by a circle, and its color represents its cluster terms. Terms with a similarity score > 0.3 kappa. The PPI network is visualized with Cytoscape.

Due to the limitations of our technical methods, we use the external standard method, which cannot accurately quantify the content of a certain fatty acid like the internal standard method. On the other hand, we first compared the PSO and FO groups to the model group. No matter if PSO or FO groups resulted in an increase in the proportion of DHA or a decrease in the proportion of AA. In our study, RNA‐seq and experimental verification confirmed that the results of the PSO group and FO group were consistent. Combined with our previous research, we demonstrated that the increase in DHA was significantly correlated with neuronal apoptosis and cognitive function (Zhang et al. 2022). In addition, particularly in the brain, DHA‐derived specialized pro‐resolving mediators (SPMs) have important anti‐inflammatory and pro‐dissolution properties and are considered to play a potential positive and important role in the occurrence and development of neurodegenerative diseases (Beyer et al. 2023). Then we found that *App*'s gene expression was not changed, but *MAPT*'s gene expression was decreased, induced by *Pparg*. The mechanism of this research indicates that n‐3 PUFA activated the PPARs/RXRs signaling pathway and RA metabolism and inhibited AA metabolism, neuroinflammation. These DEGs are enriched and clustered in omega‐3/omega‐6 FA synthesis, the PPAR signaling pathway, and the fatty acid metabolic process by PPI (Figure 6B).

We also predicted the potential transcription sites of the *MAPT* gene by *Pparg* and *Rxra* TFs using the JASPAR database and UCSC genome browser. The Pparg::Rxra's sequence motif was shown in Figure S5A by the JASPAR database. Transcription factor binding sites (TFBS) (5–20 bp) have no function in themselves and only play a role after being recognized by TFs (Wasserman and Sandelin 2004). Therefore, we need to find and predict the reliability of TFBS. At first, the promoter region of *Mapt* (2000‐start bp to 100‐end bp) was selected and has tissue specificity (brain pericyte and neuron cell), with a total four potential and possible TFBS when we screened relative scores of more than 0.80 (Figure S4B; *p* < 0.01) with reference to the previous report (Lis and Walther 2016). Unfortunately, we seem to find these critical binding sites; there is still a lack of sufficient conditions to perform validation experiments.

Interestingly, although both fish oil and peony seed oil are rich in n‐3 PUFA decreased neuroinflammation and inhibited the biosynthesis and metabolism process of AA, the related genes seem to be different. The peony seed oil promotes the n‐3 PUFA's elongation, while fish oil promotes the process of β‐oxidation. In addition, fat‐soluble vitamins and fatty acids' complex relationship was still not clear. We analyzed the relative content of fat‐soluble vitamins in different oils compared to the daily diet: peony seed oil, olive oil and lard oil rich in vitamin E; both fish oil and lard oil‐rich in vitamin D_3_, and vitamin A (VA) (Figure S6A; *p* < 0.05). RA is the bioactive metabolite of VA, which is a potential signaling molecule in the brains of growing and adult animals. Dietary supplementation can improve learning and memory in VA deficiency rodents and ameliorate cognitive declines associated with normal aging (Olson and Mello 2010; Endres 2019). In our research, lard oil is also promoting RA metabolism due to being rich in VA and seems to be positive for neuroprotection. Although some in vivo studies indicated lard oils conferred unhealthy effects (Mendiola‐Precoma et al. 2017), it's also been considered beneficial due to SFA and MUFA tending to be balanced and rich in vitamins, which could promote absorption of fat‐soluble vitamins and play an important role in the maintenance of organ function (Niki and Traber 2012). Our research suggests that peony seed oil rich in ALA can further synthesize DHA in mice, which has the same effect as direct supplementation of DHA. Both promote the synthesis of DHA in the brain, which is consistent with another study, suggesting that n‐3 PUFAs mainly produce DHA in the body in response to aging and are important for brain health (Wei et al. 2023).

## Author Contributions

**Tianyu Zhang:** conceptualization (lead), project administration (lead), resources (lead), software (lead), writing – review and editing (lead). **Ying Zhang:** investigation (supporting), methodology (supporting), project administration (supporting), writing – original draft (supporting). **Andong Ji:** software (supporting), supervision (supporting), validation (supporting), visualization (supporting). **Runjia Shi:** data curation (supporting), formal analysis (supporting), investigation (supporting), methodology (supporting). **Huiying Li:** conceptualization (equal), resources (equal), visualization (equal). **Qiangcheng Zeng:** funding acquisition (lead), investigation (lead), writing – review and editing (lead).

## Conflicts of Interest

The authors declare no conflicts of interest.

## Supporting information


Appendix S1.

## Data Availability

The data that confirm the results of this research are available from the corresponding author upon reasonable request.
