# Correction to “Peony Seed Oil Inhibited Neuroinflammation by PPAR/RXR Signaling Pathway in D‐Gal Induced Mice”

**DOI:** 10.1002/fsn3.70158

**Published:** 2025-05-08

Zhang, T. , Zhang, Y. , Ji, A. , Shi, R. , Li, H. and Zeng, Q. (2025), Peony Seed Oil Inhibited Neuroinflammation by PPAR/RXR Signaling Pathway in D‐Gal Induced Mice. Food Sci Nutr, 13: e70000. 10.1002/fsn3.70000 40018014 PMC11866050

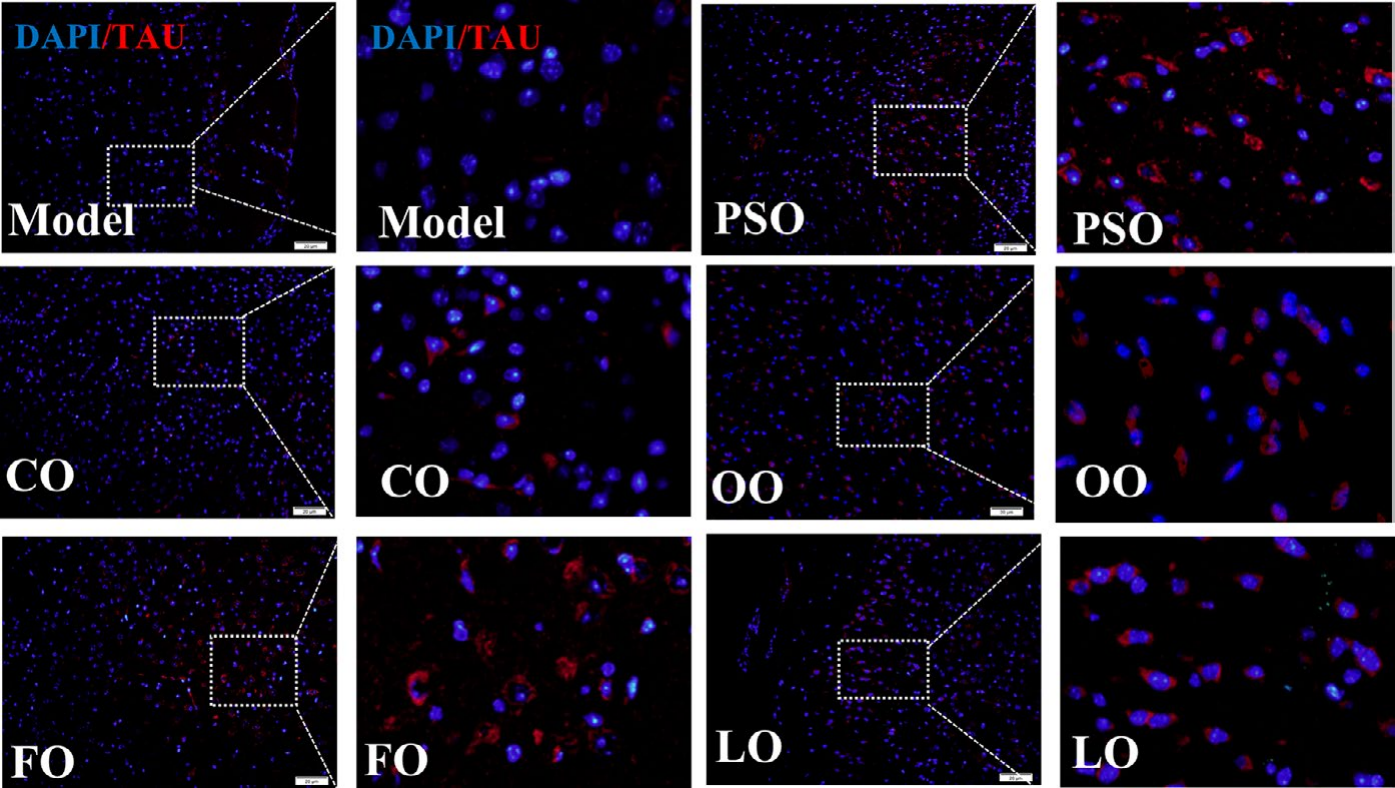

In Figure 5D, the images for the OO group were incorrect. Here is the corrected figure:

We apologize for this error.

