# Supplementary material for: Peony Seed Oil Inhibited Neuroinflammation by PPAR/RXR Signaling Pathway in D‐Gal Induced Mice
Source: Food Sci Nutr. 2025 Feb 27;13(3):e70000. doi: 10.1002/fsn3.70000 (PMC11866050; doi:10.1002/fsn3.70000)
Supplement: Supplementary file 1 — Appendix S1. [file FSN3-13-e70000-s001.docx]

**Supplementary materials**

**Peony seed oil inhibited neuroinflammation by PPAR/RXR signaling pathway in D-gal induced mice**

Ying Zhang^1, †^, Huiying Li^2,^ ^†^, Andong Ji^3^, Runjia Shi^3^,Tianyu Zhang^1,3,*^, Qiangcheng Zeng^1,*^,

**Author affiliations:**

*^1^**College of Life Sciences, Dezhou University, Dezhou, Shandong, China.*

*^2^Department of Public health,The Third People's Hospital,Jinan,China*

*^3^Institute of Nutrition and Health, Qingdao University, Qingdao, China.*

| Gene name | Sequence (5'->3') | Genebank Accession |
| --- | --- | --- |
| *Acox1* | F:CCGCCACCTTCAATCCAGAG  R:CAAGTTCTCGATTTCTCGACGG | NM_015729 |
| *Acox3* | F:CAGAATGGTGTGCTAGAGCGT  R:AGCCTGTCGGCTACAGATTTG | NM_030721 |
| *Fads1* | F:AGCACATGCCATACAACCATC  R:TTTCCGCTGAACCACAAAATAGA | NM_146094 |
| *Fads2* | F:TCATCGGACACTATTCGGGAG  R:GGGCCAGCTCACCAATCAG | NM_019699 |
| *Elovl2* | F:CACGTACCTGCTCTCGATATGG  R:TGTGATTGCGAGGTTATACAAGG | NM_019423 |
| *Elovl5* | F:CAGATCACCGTGCTCCATGTC  R:CTGTTGAGTGTCGCACCAAA | NM_134255 |
| *Actb* | F:GGCTGTATTCCCCTCCATCG  R:CCAGTTGGTAACAATGCCATGT | NM_007393 |

Table. S1 Information of Primers

F:Forward primer; R: Reverse primer

Fig S1


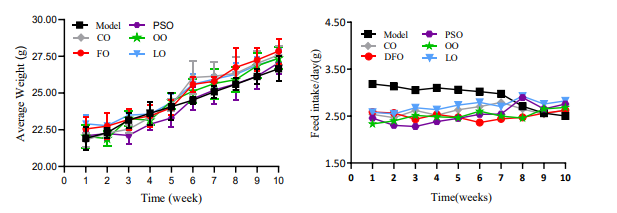


Fig S1 Average feed intake and weight were been recorded statistically. Data are presented as mean ± SD; *P < 0.05. **P < 0.01, a significant difference compared to the Model group determined by the t-test. Six mice that were sequenced per group.

Fig S2


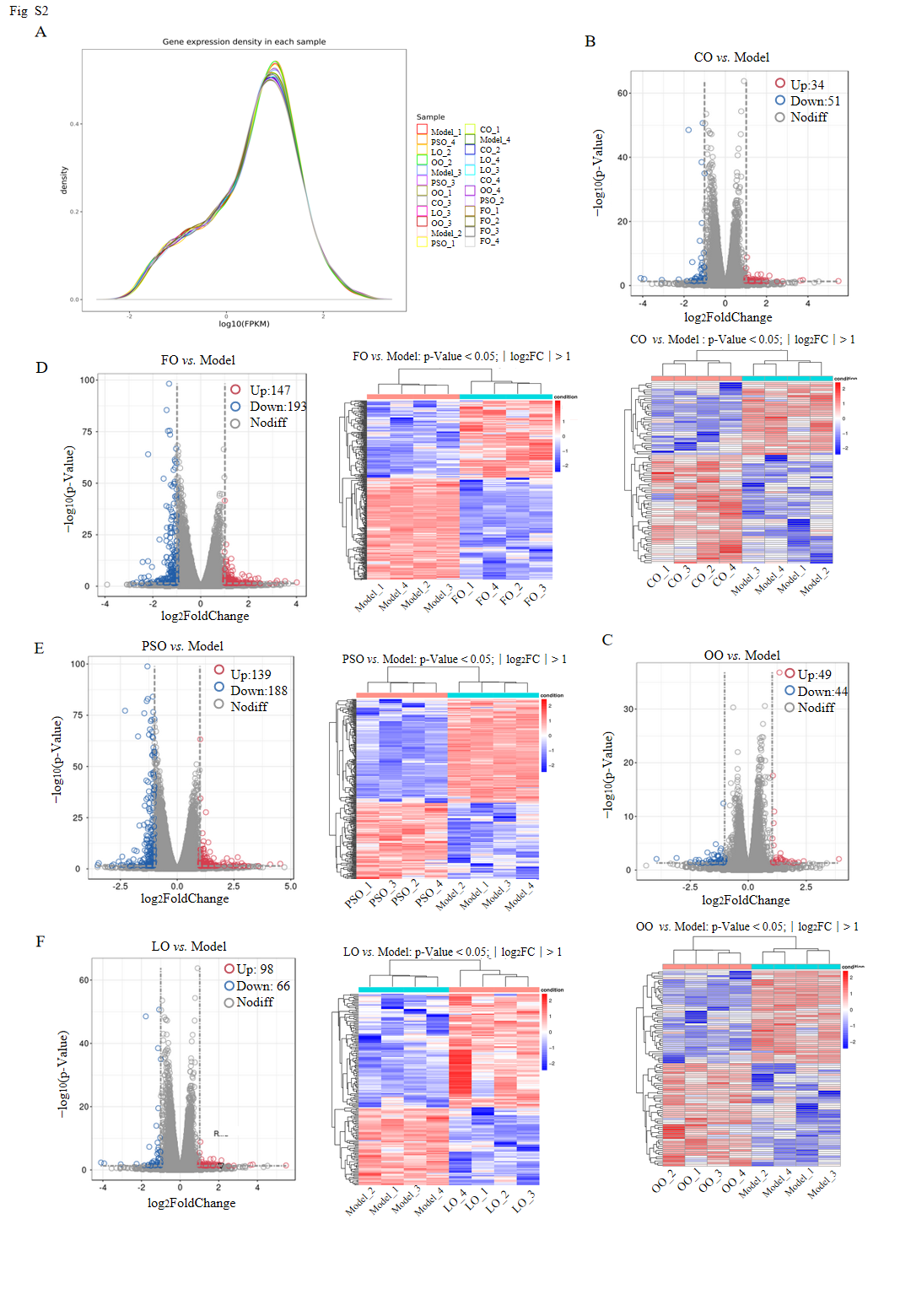


Fig S2 (A) Density distribution curve of FPKM value of each sample. The curves with different colors in the plot represent different samples, the x-axis of the points on the curve represents log_2_FPKM of the samples, and the y-axis of the points represents the probability density. (B) Volcano maps showed the DEGs in treated groups compared to the Model group. Dot’s color indicates genes up-regulated (red), down-regulated (blue), or not different (grey). Red indicates relatively high expression DEGs, and blue indicates relatively low expression DEGs showed in the heatmap. DEGs were identified by *P*-value＜0.05, |Log2FC|>1. Data are representative of four replicates that were sequenced, respectively.

Fig S3


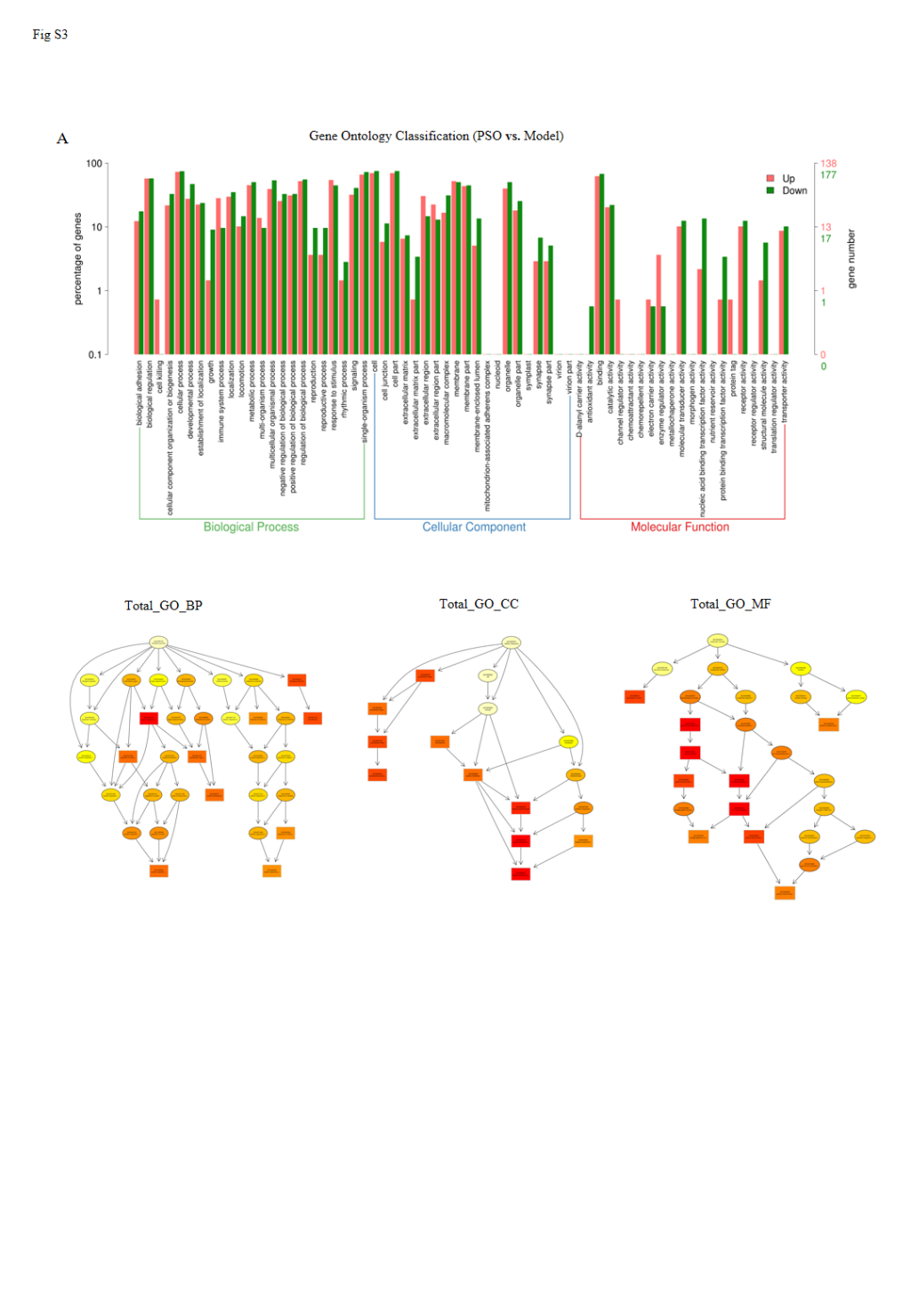


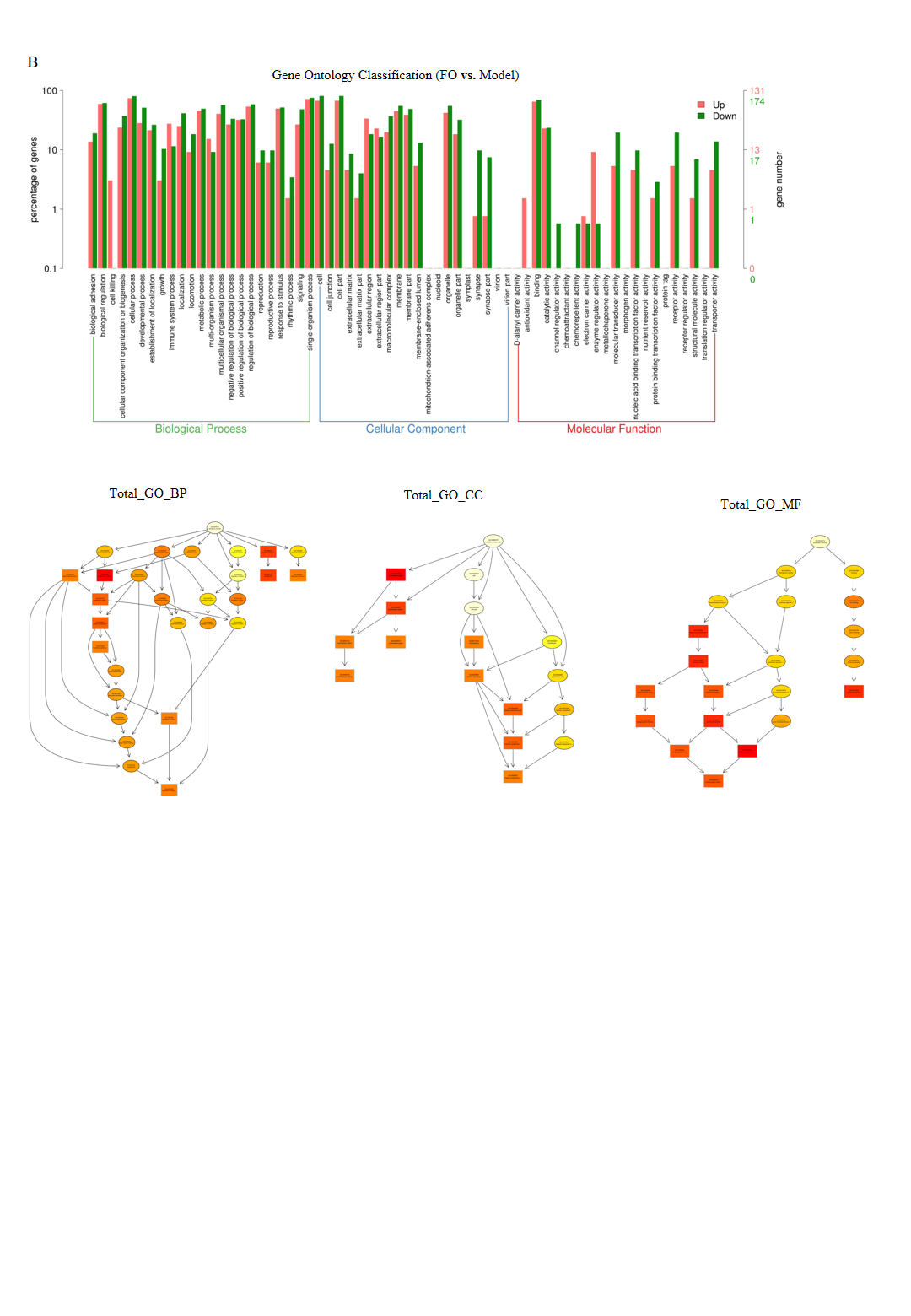


Fig S3 (A, B) Comparison of up-DEGs (red) and down-DEGs (green) at GO Level2 in FO and PSO groups *vs.* the Model group. DEGs’ topGO graph provides a visual representation of the enriched GO terms and their relationships. Enrichment analysis for each GO term Colors of rectangles represents enrichment significance, increasing significance from yellow to red. DEGs were identified by *P*-value＜0.05, |Log2FC|>1. Data are representative of four replicates that were sequenced, respectively.

Fig S4


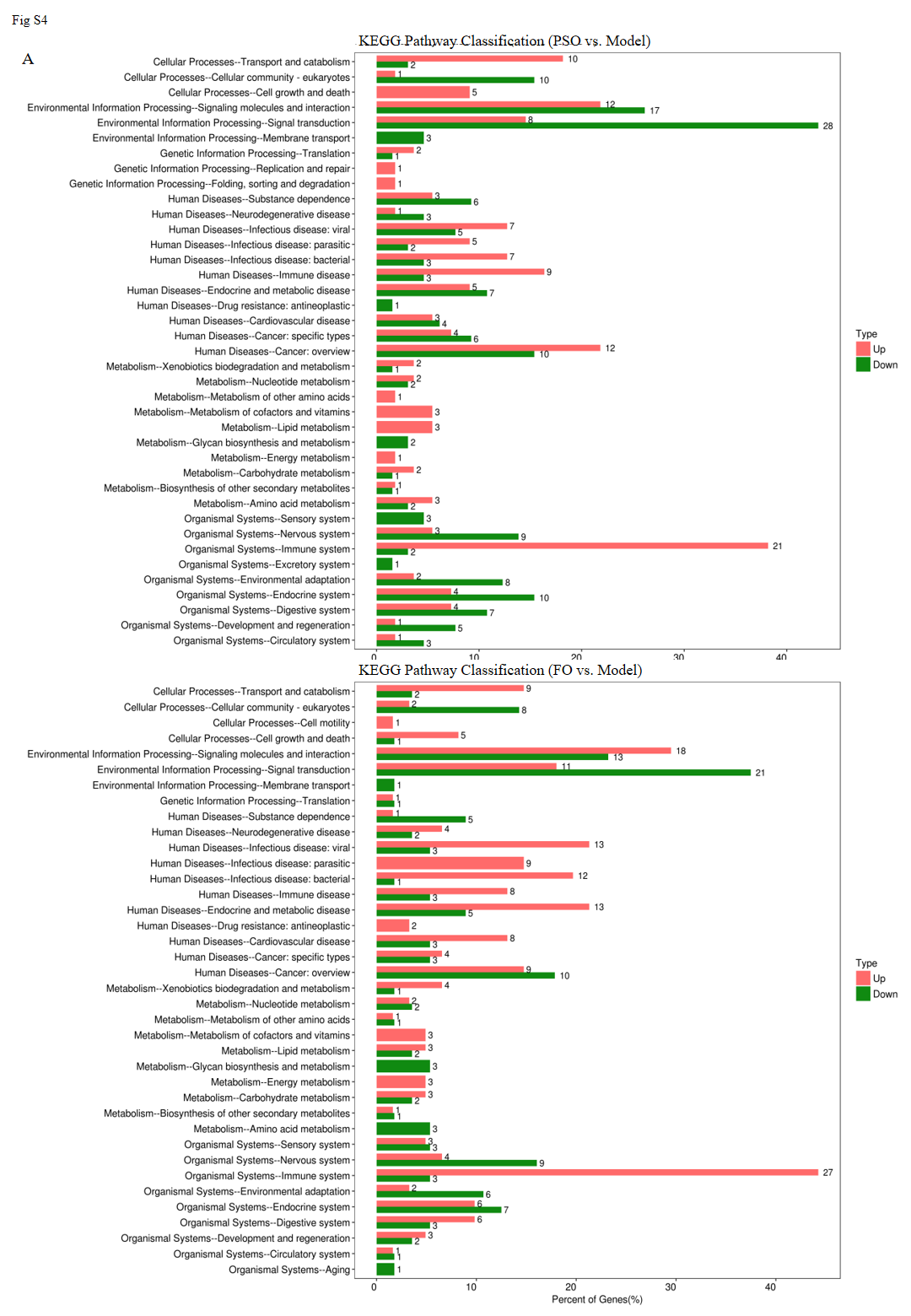
 Fig S4 Comparison of DEGs at KEGG Level2 classification in FO and PSO groups vs. the Model group. The x-axis is the enrichment score of upregulated (red), downregulated (green) annotated to each pathway. DEGs were identified by *P*-value＜0.05, |Log2FC|>1. Data are representative of four replicates that were sequenced, respectively.

Fig S5


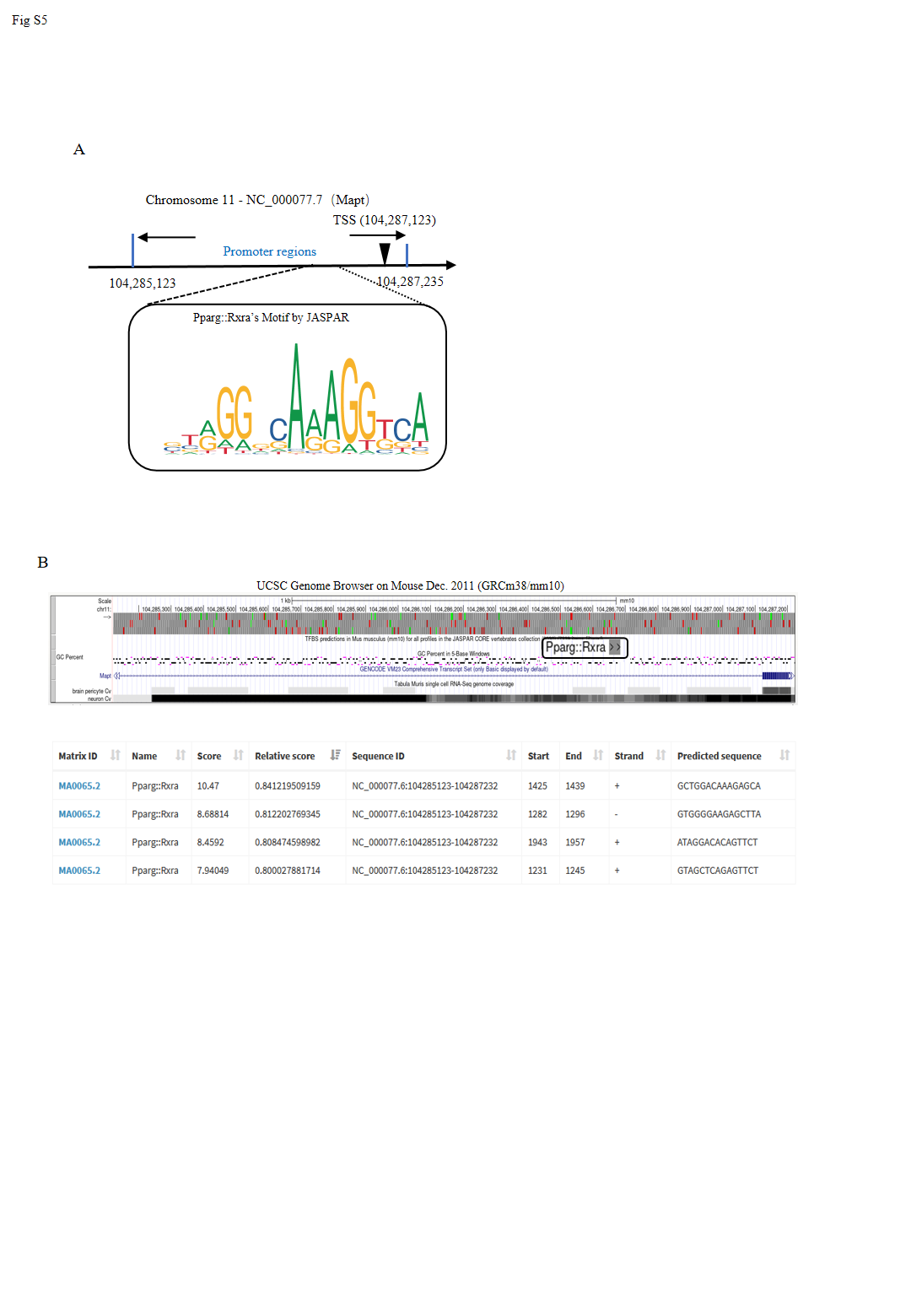


Fig S5 (A) Predicted Pparg:: Rxra binding motif site sequence from the database and mapped to predicted *MAPT*’s gene promoter regions (downstream 200 bp~ upstream 200 bp) were selected. (B) *MAPT* gene visualized in the UCSC Genome Browser and predicted TFBS regions (relative score > 0.80; *P*-value < 0.01).

Fig S6


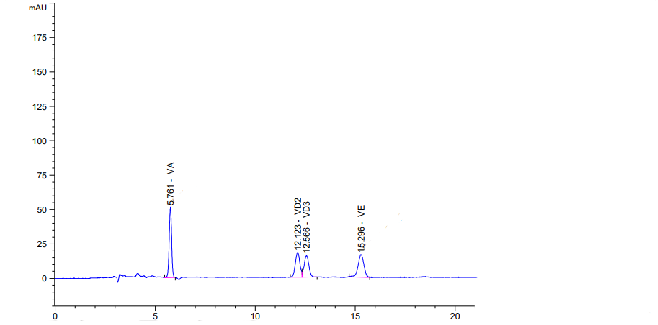


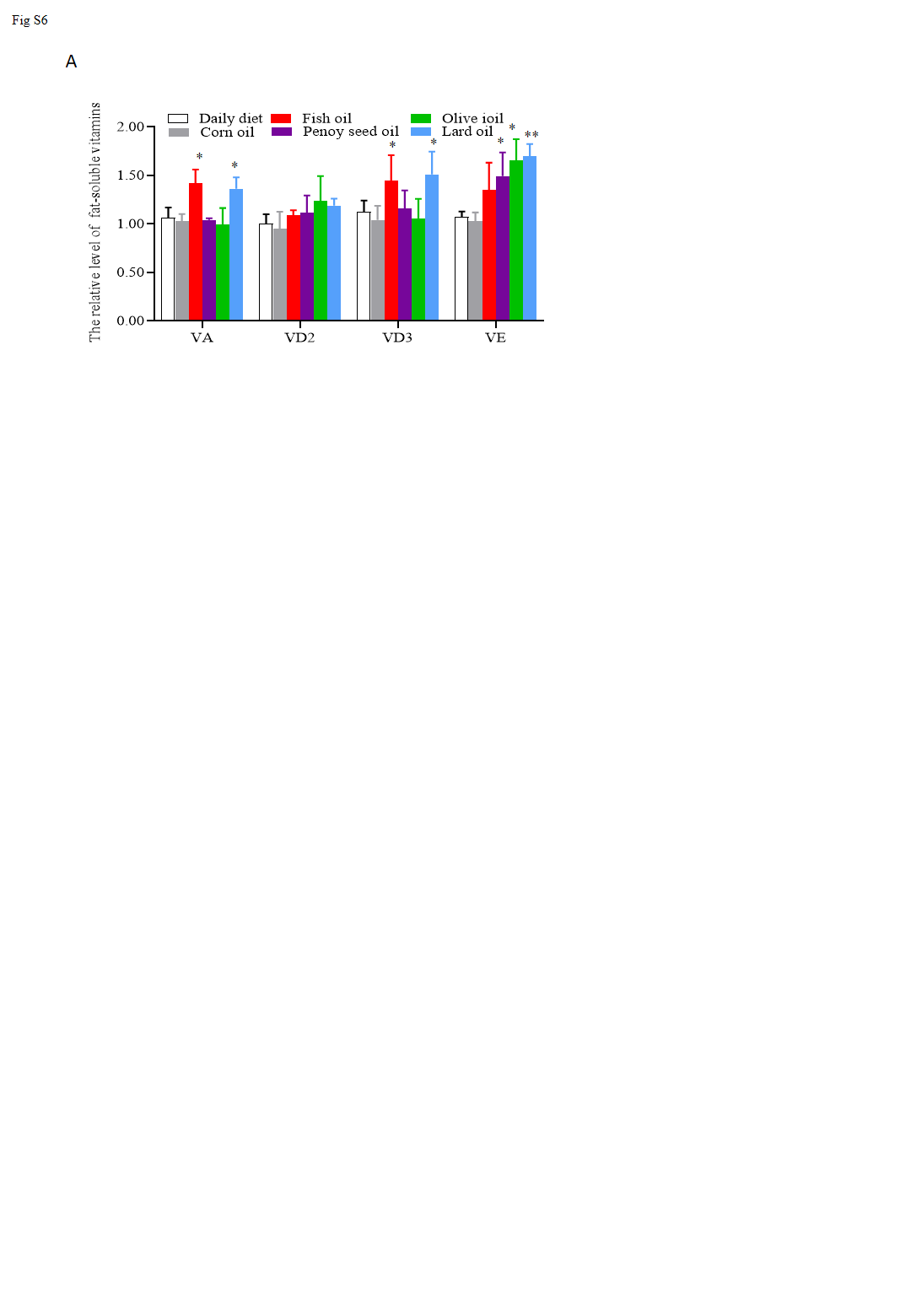


Fig S6 Determination of four fat-soluble vitamins for feed and oils by HPLC. (B) Average feed intake and weight were been recorded statistically. (C) Organ coefficients of brain, liver and fat were been calculated (organ weight/ total weight), respectively. Data are presented as mean ± SD.
